# Machine Learning: Applications and Advanced Progresses of Radiomics in Endocrine Neoplasms

**DOI:** 10.1155/2021/8615450

**Published:** 2021-10-11

**Authors:** Yong Wang, Liang Zhang, Lin Qi, Xiaoping Yi, Minghao Li, Mao Zhou, Danlei Chen, Qiao Xiao, Cikui Wang, Yingxian Pang, Jiangyue Xu, Hao Deng, Longfei Liu, Xiao Guan

**Affiliations:** ^1^Department of Urology, Xiangya Hospital, Central South University, No. 87 Xiangya Road, Changsha 410008, Hunan, China; ^2^Department of Radiology, Xiangya Hospital, Central South University, No. 87 Xiangya Road, Changsha 410008, Hunan, China; ^3^Department of Clinical Laboratory, Xiangya Hospital, Central South University, No. 87 Xiangya Road, Changsha 410008, Hunan, China; ^4^Department of Minimally Invasive Surgery, The Second Xiangya Hospital of Central South University, Changsha, Hunan 410011, China

## Abstract

Endocrine neoplasms remain a great threat to human health. It is extremely important to make a clear diagnosis and timely treatment of endocrine tumors. Machine learning includes radiomics, which has long been utilized in clinical cancer research. Radiomics refers to the extraction of valuable information by analyzing a large amount of standard data with high-throughput medical images mainly including computed tomography, positron emission tomography, magnetic resonance imaging, and ultrasound. With the quantitative imaging analysis and model building, radiomics can reflect specific underlying characteristics of a disease that otherwise could not be evaluated visually. More and more promising results of radiomics in oncological practice have been seen in recent years. Radiomics may have the potential to supplement traditional imaging analysis and assist in providing precision medicine for patients. Radiomics had developed rapidly in endocrine neoplasms practice in the past decade. In this review, we would introduce the general workflow of radiomics and summarize the applications and developments of radiomics in endocrine neoplasms in recent years. The limitations of current radiomic research studies and future development directions would also be discussed.

## 1. Introduction

Endocrine neoplasms are derived from specialized hormone-secreting cells. Most of these tumors specialize in synthesizing and secreting hormones with a small portion lacking hormone-secreting ability [1]. Except for those that occurred in classic endocrine glands, endocrine neoplasms also include multiple endocrine neoplasms, neuroblastoma, lung neuroendocrine tumors, small intestinal neuroendocrine tumors, and skin neuroendocrine tumors [2].

Endocrine neoplasms remain a great threat to human health. Breast cancer accounts for about 30% of female cancers, and its incidence rate is still rising [3]. In 2020, there would be approximately 52,890 new thyroid cancers in the USA [4]. Pancreatic cancer has a high mortality rate with an estimated 47,050 cancer deaths occurred in 57,600 new cancer cases [4]. Pheochromocytoma and paraganglioma are important causes of secondary hypertension and may lead to severe cardiovascular and cerebrovascular diseases [5]. Therefore, it is extremely important to make a clear diagnosis and timely treatment of endocrine tumors.

Imaging is widely accepted as an important and useful tool in oncologic research because of its noninvasiveness, convenience, and repeatability, which is used not only for the diagnosis and staging of tumors but also for tumor anatomical characteristics assessment and cancer management evaluation [6]. On the other hand, imaging can provide information about the overall tumor phenotype, including the heterogeneity within the tumor [7]. However, considering that imaging features are often visually observed and qualitatively described by radiologists, these visual assessments are not always consistent within and between observers [8]. Therefore, it is necessary to objectively and repeatedly quantify various imaging features that may have the potential to reveal the underlying biological mechanisms of tumors [6]. Radiomics refers to the extraction of valuable predictive information by analyzing a large amount of high-throughput medical images [9]. With subsequent data analysis and model building, radiomics can reflect specific underlying characteristics of a disease that otherwise could not be evaluated visually, which may supplement traditional imaging analysis and assist in providing precision medicine for patients.

More and more promising results of radiomics in endocrine neoplasms have emerged in recent years. In this review, we would introduce the general workflow of radiomics and summarize the applications and developments of radiomics in endocrine neoplasms. The limitations of current radiomic research and future development directions would also be discussed.

## 2. The Basic Principle and Workflow of Radiomics

Radiomics is based on the hypothesis that quantitative analysis of tumors by numerous radiomic features can obtain valuable predictive information [10, 11]. These radiomic features, including the shape, size or volume, intensity, and texture of the tumor phenotype, are different from or complementary to that provided by clinical reports, laboratory tests, genomics, or proteomics analyses [12]. The purpose of radiomics is to explore and employ these radiomic features combined with other useful information to design models for the overall management of the disease and help implement clinical decisions and improving treatment choices [13].

**Figure 1:**
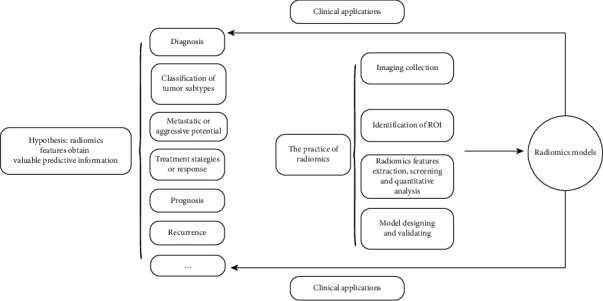
The workflow and applications of radiomics.

The practice of radiomics involves four main steps: (1) imaging collection; (2) segmentation of the region of interest (ROI); (3) radiomic features extraction, screening, and quantitative analysis; and (4) model designing and validating (Figure 1).

### 2.1. Image Collection

Images include CT/PET/MRI/US, and tumor specificity imaging exams could be used for radiomic analysis. It is normal that imaging protocols and scanners vary between medical centers. It may not be a problem when it comes to the traditional assessment of imaging features. However, in radiomics, these differences may introduce changes unrelated to underlying biological effects [13]. Thus, preprocessing of original images is usually required before feature extraction.

### 2.2. Segmentation of ROI

ROI segmentation is a key step in radiomics as it defines the area of the image from which radiomic features are extracted. In mostly radiomic studies, ROI was manually identified by experienced radiologists [14]. However, this method may introduce high interobserver variability, which can produce unstable radiomic features [15]. Two or more segmentations can be conducted on the same lesion, and correlation analysis could be used to identify feature stability [16]. Methods of automatic and semiautomatic segmentation were also reported in a number of studies, which might potentially reduce the influence of interobserver variability.

### 2.3. Radiomic Features Extraction, Screening, and Quantitative Analysis

By extracting, screening, and quantitatively analyzing radiomic features, also known as quantitative imaging biomarkers, we can acquire a wealth of predictive information including diagnosis, prognosis, or tumor microenvironment. Among these features, deep features are suitable to map nonlinear representations when there are sufficient training data cases. However, the applications of deep features are still under debate due to their low interpretability and difficulty in conceptualizing [15].

### 2.4. Radiomic Model Designing and Validating

Three aspects are usually involved in radiomic model building: feature selection, modelling methods, and model validation. A huge number of radiomic features would be generated in the process of radiomic analysis. Thus, it is of great importance to select features to avoid overfitting. The selection of methods for analysis depends on several factors, including sample size and the applications of radiomic measurements. There exist many statistical methods and machine learning (ML) algorithms for radiomic analysis. Model validation is used to evaluate the performance and applicability of the radiomic model. Internal and/or external validation should be performed to ensure the generalizability of the model to all of the targeted patients. The receiver operating characteristic (ROC) curve and the area under the ROC curve (AUC) are usually used to calculate the performance of the model.

## 3. Radiomics in Endocrine Neoplasms

### 3.1. Pituitary Adenomas

#### 3.1.1. Diagnosis and Tumor Subtypes Classification

Pituitary adenomas (PAs) are common types of intracranial tumors with a prevalence of 80 to 100 cases/100,000 persons and an annual incidence of 4 cases/100,000 persons [17–19]. Early and accurate diagnosis is important for patients with PAs. Based on MRI, Zhang et al. conducted a study aiming to differentiate pituitary adenoma from the Rathke cleft cyst, and the results showed that two radiomic features had promising and practical values in distinguishing those two tumors, with an AUC of more than 0.75 [20]. The subtype of PAs plays a major role in determining subsequent treatment. Traditionally, the subtype can only be determined by immunohistochemical staining after surgery. A recent study by Peng et al. revealed that an MRI-based radiomic model could be used to predict immunohistochemical results of pituitary adenoma preoperatively (with an accuracy of 0.89 and an AUC of 0.9549) [21]. Besides, MRI-based radiomic features had a great potential to differentiate between nonfunctional subtypes and other subtypes of pituitary adenomas preoperatively [22].

#### 3.1.2. Aggressiveness

Predicting pituitary tumor behavior preoperatively remains a clinical challenge because no valid factor has been determined. PAs with a high Ki-67 proliferative index have been considered to be a high risk of aggressiveness [23]. Ugga et al.'s study found that MRI-based radiomics could indirectly predict tumor aggressiveness by predicting Ki-67 proliferative index in pituitary macroadenomas, with excellent accuracy of more than 91% [24].

#### 3.1.3. Treatment Strategies and Response

Surgery is the first-line therapy for most pituitary macroadenomas, but once the tumor invades the cavernous sinus, it becomes a clinical concern due to different surgical strategies. On the other hand, preoperative assessment of PAs consistency and vascularity is of equal significance for surgical strategies and risk evaluation. Predicting the cavernous sinus invasion preoperatively using MRI-based radiomic methods was proved to be an effective method with an AUC of 0.899, which contributes to surgical strategies decisions [25]. The prediction of treatment response before surgery is important for making personalized treatment strategies for patients with invasive functional pituitary adenoma (IFPA). Fan et al. conducted a study based on preoperative MRI, aiming at predicting the treatment response of patients with IFPA. The result showed that both primary and validation models both achieved good results with an AUC of more than 0.8.

#### 3.1.4. Prognosis

Using traditional scoring systems to predict postoperative outcomes is challenging because of the existence of heterogeneity, which means an individual patient has different risk factors of prognosis. Hollon et al. used a machine learning approach to predict early outcomes after surgery, and the result based on MRI radiomics showed a specificity of 93.3% and an accuracy of 87.0%, indicating that early postoperative outcomes of PAs could be assessed by a radiomic approach [26].

In this section, we summarized the applications of radiomics in PAs, including diagnosis and classification of tumor subtypes, evaluation of tumor aggressiveness, selection of treatment strategies, response to treatment, and prognosis. It is not strange that the applications of radiomics in PAs are mainly based on MRI as it remains the most important imaging modality in the management of PA. Although most studies proposed promising clinical applications, reproducibility, robustness, and generalizability were the major limitations of current research [27] (Table 1).

### 3.2. Thyroid Cancer

#### 3.2.1. Diagnosis

It is important to make an early diagnosis of thyroid cancer in order to avoid overtreatment in patients with low-risk diseases. Thyroid incidentalomas are difficult to diagnose due to the lack of matching symptoms in the patients. A previous study had demonstrated that ^18^F-FDG-PET/CT texture analysis seemed to be a promising method to predict the final diagnosis of thyroid incidentalomas (with an unsatisfied AUC of 0.66) [29]. But it still needs further validation in larger subsequent studies.

#### 3.2.2. Metastasis

Papillary thyroid carcinoma (PTC) is the most common histology type of thyroid malignancy [30–32], which has a high incidence rate and is often overdiagnosed and overtreated clinically. Lymph node metastasis is the most important risk factor associated with recurrence and low survival in PTC patients [33]. Preoperative prediction of lymph node metastasis or aggressiveness in patients with PTC can improve surgical planning and reduce the risk of surgery. Liu et al. constructed radiomic models using US images to predict the lymph node metastasis of PTC preoperatively, and the result achieved an AUC of 0.782 and an accuracy of 0.712 [34]. Similarly, Wang et al. showed that the accuracy of the US-based radiomic method was much higher than that of the US examination in the prediction of metastasis of PTC [35]. According to Song et al.'s study, DWI-based radiomics may have the potential to differentiate benign from malignant thyroid nodules with an outstanding AUC of 0.97 [36]. On the other hand, Yoon et al. used US radiomic methods to predict the BRAF^V600E^ mutation status that was associated with aggressive clinical behavior, demonstrating that radiomic features limitedly predicted clinical aggressive behaviors values as noninvasive biomarkers [37].

Radiomics had been widely applied in the metastasis prediction in thyroid cancer. Many studies had shown that preoperative noninvasive radiomics could be used to assess the risk of PTC lymph node metastasis and guide surgeons to make clinical decisions [38–40].

#### 3.2.3. Treatment Strategies

In terms of selecting suitable surgical strategies, it is important to determine the occurrences of extrathyroidal extension (ETE) in patients with PTC. Chen et al. designed a CT radiomic model to predict ETE preoperatively in patients with PTC. The result had an adaptive AUC of 0.837 [41].

#### 3.2.4. Prognosis

Most thyroid cancers are treatable and have a relatively favorable survival rate, but a small portion of PTC have aggressive clinical behavior and patients with PTC may recur or die due to this disease. Thus, the prediction of the prognosis should not be ignored. Park et al. explored the connection between radiomic features and disease-free survival (DFS) based on US radiomics, and the result demonstrated that radiomic features were significantly associated with DFS [42] (Table 2).

Imaging examinations for applications of radiomics in thyroid cancers consist of 18F-FDG-PET/CT, US, CT, and MRI. These models were used in multiple aspects of oncologic practice in thyroid cancers. Notably, the reliability of the predictive performance and clinical applications may be decreased because of discussing the predictive value of radiomics itself without considering the influence of clinical information, such as therapy strategies and tumor stages. In addition, the ethical issues regarding the use of radiomics in patient stratification and treatment response-based prognosis should also be treated with caution [43].

### 3.3. Breast Cancer

#### 3.3.1. Tumor Risk Assessment

Previous studies had proved that the risk of breast cancer was strongly related to mammographic parenchymal patterns, especially when it is assessed by percent mammographic density. In order to establish personalized screening recommendations and preventive strategies, the assessment of the risk of developing breast cancer has become more and more important today [44]. Yan et al. built a new bilateral mammographic density segmentation method based on mammography to improve the accuracy of breast cancer prediction, and the results showed an adaptive AUC of 0.83 and an accuracy of 81% [45]. Similarly, the study by Kontos et al. achieved an AUC of 0.84, demonstrating that radiomics had the potential to predict breast cancer risk factors [46]. Pinker et al. declared that radiomic phenotypes could assess mammographic parenchymal complexity and could provide additional useful information for risk assessment beyond breast density [47].

#### 3.3.2. Diagnosis

Early diagnosis and timely treatment are critical to reducing cancer mortality in patients with breast cancer. Although previous reviews had summarized the applications of radiomics in the diagnosis of breast cancer [48], various studies were investigated for further exploration and validation. Ji et al. used an MRI-based radiomic method to explore its potential in distinguishing between malignant and benign breast lesions; the results achieved an AUC of 0.88 and a sensitivity of 99.5% [49]. According to Wang et al.'s study, triple-negative (TN) breast cancers were identified using MRI-based radiomics, achieving an excellent AUC of 0.878 [50]. In Lee et al.'s study, US texture features showed potential application in differentiating TN breast cancer from fibroadenoma [51].

Studies with MRI-based radiomics had shown that different radiomic parameter values were displayed in different breast tissues, and malignant tissues were obviously different from other tissues [52, 53]. In a study of US-based radiomics, some radiomic features may help distinguish benign breast tumors from malignant ones [54]. According to Luo et al., US radiomics was potentially useful for predicting breast malignancy (with an AUC of 0.928) [55]. Researchers had also utilized the characteristic digital breast tomosynthesis to assess its relationship with malignancy; the result had limited values [56]. Yu et al. proved that mammography features could aid in diagnosis in patients with TN breast cancer [57]. All of the above studies indicated that radiomic approaches had the potential to predict malignancy, which was helpful in the detection and diagnosis of breast cancer [58].

#### 3.3.3. Molecular Typing Classification

Breast cancer patients with different immunohistochemical (IHC) subtypes have diverse clinical outcomes and responses to therapy. It is critical to identify the subtypes in terms of selecting appropriate personalized therapy and predicting therapeutic response [59, 60]. Xie et al. developed MRI-based radiomic methods to classify the subtype of breast cancer, finding that the radiomic model had an accuracy of 91.0% in distinguishing between triple-negative tumors and nontriple-negative tumors [61]. Fan et al. combined clinical information with MRI-based radiomics to predict the molecular subtypes of breast cancer. The results showed that radiomic models had excellent performance in discriminating subtypes of breast cancer [62]. Wu et al. got a similar conclusion by using MRI radiomics [63]. BEng et al. found that an MRI-based radiomic model combining peritumoral and intratumoral radiomic features had the potential to identify the HER2-E subtype (AUC, 0.89) [64]. Results of several similar studies also indicated that radiomic features were potential biomarkers to distinguish four molecular subtypes of breast cancer [65–67].

#### 3.3.4. Metastasis

An accurate assessment of axillary lymph node (ALN) metastasis is important for choosing therapy strategies and predicting prognosis in early-stage breast cancer [68]. Zheng et al. developed a US-based radiomic model to predict ALN metastasis in early-stage breast cancer. The model showed an excellent AUC of 0.902 in distinguishing disease-free axilla and any axillary metastasis [69]. Other US-based radiomic methods achieved an approving AUC of more than 0.9 in predicting the ALN metastasis of breast cancer [70]. A mammography-based radiomic model designed by Yang et al. predicted the ALN metastasis preoperatively with an AUC of 0.895 in the training cohort and an AUC of 0.875 in the validation cohort [71]. Dong et al. conducted an MRI radiomic study to predict the metastasis of sentinel lymph nodes in patients with breast cancer. A maximum AUC of 0.863 was achieved, providing a potential noninvasive approach in clinical practice [72]. All these studies indicated that radiomic models were reliable for predicting ALN metastasis in patients with early-stage breast cancer preoperatively.

#### 3.3.5. Treatment Response

In the field of precision medicine for breast cancer, the prediction of treatment response is the focus of disease management [73]. Neoadjuvant chemotherapy (NAC) is the first-line treatment for advanced local breast cancer as it reduces tumor volume and the risk of distant metastasis before surgery [74]. Tahmassebi et al. constructed an MRI radiomic model to predict the response of patients with breast cancer to NAC and achieved a stable performance with high accuracy (with an AUC of 0.92) [75]. BEng et al. explored to determine whether MRI-based radiomic features could estimate responses to NAC in HER2-positive breast cancer patients. The result demonstrated that radiomic features were significantly associated with response to NAC, indicating that radiomics had the potential to predict the response to HER2-targeted therapy [64]. According to Braman et al.'s study, textual analysis of peritumoral and intratumoral regions achieved a maximum AUC of 0.78 in predicting pathological complete response to NAC [76]. Based on mammographic radiomic features, Yu et al. aimed to investigate the level of tumor-infiltrating lymphocytes in TN breast cancer. The result revealed that mammographic features had the potential to be an imaging biomarker in predicting response to NAC [57].

Henderson et al. revealed that MRI-based interim heterogeneity changes were particularly associated with pathologic complete response to NAC with an AUC of 0.845 [77]. Similarly, Sutton et al. proposed to classify pathologic complete response in breast cancer patients after NAC. The result achieved a maximum AUC of 0.83, indicating that MRI radiomic models had the potential to assess pathologic complete response to NAC [78].

#### 3.3.6. Prognosis and Recurrence

Breast cancer is widely known as a heterogeneous disease. The current major prognostic factors of breast cancer include lymph node metastasis, obesity, Ki-67 index, pathologic complete response, and tumor volume [79, 80]. Obeid et al. aimed to assess the correlations between peritumoral fat and MRI-based radiomic features. The results indicated that peritumoral fat and BMI >30 were significantly correlated with radiomic features [81]. Studies also revealed that MRI-based radiomic approaches could predict the expression of Ki-67 [82, 83]. Drukker et al. showed that MRI radiomic features contributed to the prediction of recurrence-free survival (RFS) in NAC treatment of breast cancer [84]. Basing on MRI radiomics, Wu et al. found that radiomic features were independent prognostic factors beyond traditional risk predictors [85]. Dietzel et al.'s study demonstrated that radiomic models based on MRI improved the survival prediction in primary breast cancer [86]. What's more, HER2 protein overexpression was defined as an aggressive subtype associated with poor clinical outcomes [87]. In a study by Yang et al., radiomics could assess prognosis through predicting HER2 status [88].

According to Li et al., there was a significant association between MRI radiomic features and multi-gene assay recurrence score (*P* < 0.001), proving that radiomics was useful to assess the risk of breast cancer recurrence [89]. Tokuda et al. conducted a study to examine the correlation between MRI radiomic features with a 95-gene classifier for recurrence prediction in patients with estrogen receptor (ER) positive breast cancer. The study showed promising results [90]. Nam et al. investigated the correlations between MRI radiomic features and Oncotype DX recurrence scores in patients with ER-positive breast cancer. An AUC of 0.759 was achieved in discriminating low from non-low OD risk groups in ER-positive invasive breast cancers [91].

Radiomics has been applied in almost every aspect of breast cancer management. Other “omics” studies, including genomics, transcriptomics, proteomics, and metabolomics, are also utilized to characterize the molecular biology of tumors in recent years. However, the association between these “omics” technologies and radiomics in breast cancer is not very clear and needs to be explored in further researches. Better precision medicine for breast cancer may be achieved by integrating quantitative information of clinical, histological, and these omics data.

### 3.4. Pancreatic Neuroendocrine Tumors

#### 3.4.1. Tumor Subtypes Classification

Pancreatic cystic neoplasms include serous cystic neoplasms, intraductal papillary mucinous neoplasms (IPMNs), mucinous cystic neoplasms (MCNs), and solid pseudopapillary neoplasms. Most pancreas serous cystic neoplasms are benign with a low risk of metastasis and do not require surgical treatment [92, 93]. However, the other three types of pancreatic cystic neoplasms have a distinct ability to become malignant and are recommended for surgical treatment [94]. Therefore, it is important to correctly diagnose serous cystic neoplasms preoperatively in order to avoid unnecessary surgeries. However, the previous study had shown that the diagnostic accuracy of cyst fluid analysis and imaging in serous cystic neoplasms was low and unsatisfactory [95]. A new method is of an urgent need to determine the nature of pancreatic cystic neoplasms before surgery.

Radiomics had been used to diagnose pancreas serous cystic neoplasms preoperatively. According to Shen et al., CT-based radiomic classifiers had the potential to differentiate serous cystadenoma from IPMN and MCN preoperatively [96]. Two previous CT-based radiomic studies had shown that radiomics could predict the malignant potential of IPMNs and had important application values in making a clinical decision [97, 98]. Clinicians correctly diagnosed only 31 of 102 cases of serous cystic neoplasms, while CT-based radiomic methods achieved a sensitivity over 65% and a specificity over 70% in a recent study, which had improved diagnostic accuracy and helped clinicians making better decisions [99]. However, it would lead to misdiagnosis inevitably, which may limit the applications of radiomics in this field. Another similar study also provided preliminary evidence that CT radiomics may aid in the differentiation of pancreatic serous cystadenomas from mucinous cystadenomas, but multicenter studies with larger samples validation were still needed [100].

#### 3.4.2. Metastasis

More than 80% of patients have metastases due to the lack of proper early diagnostic methods. Preoperative identification of lymph node involvement is important to evaluate prognosis and decide individualized treatment strategies. However, pathological specimens are usually obtained after surgery. For this reason, two recent studies were conducted to explore whether radiomics could predict lymph node metastasis preoperatively. The results showed that preoperative CT-based radiomics was significantly associated with the risk of lymph node metastasis [101, 102].

#### 3.4.3. Treatment Response

Chemoradiotherapy has been widely used in locally advanced pancreatic cancer (LAPC) [103]. It will play a critical role in the management of LAPC patients in the future [104]. For this reason, prediction of posttreatment response could help select patients who would benefit most from chemoradiotherapy. Two recent studies had found important changes in CT radiomic features that could be used to assess the posttreatment response to radiotherapy for pancreatic cancer [105, 106]. According to Parr et al., CT-based radiomic models were better to predict treatment outcomes (survival or recurrence) than those of clinical features [107]. What's more, Nasief et al.'s study showed that combining CT radiomics with CA19-9 (which was widely accepted as a clinical biomarker for pancreatic cancer) could improve the ability to predict posttreatment response [108].

Pancreatic ductal adenocarcinoma (PDAC) accounts for the majority of pancreatic cancer [109]. Immunotherapy has become one of the main treatments for PDAC in recent years [110]. Studies had shown that the dendritic cell (DC) based cancer vaccines could effectively reduce tumor-specific T-cell effector in PDAC patients [111]. An MRI-based radiomic study showed that radiomics could serve as an imaging biomarker for early immunotherapy response assessment in a KPC transgenic mouse model of PDAC [112]. All of these demonstrated the potential ability of radiomics to predict treatment response in pancreatic cancer.

#### 3.4.4. Prognosis

Detection combined with treatment at the precursor lesions stage contributes significantly to the reduction of morbidity and mortality. Lymph node metastasis and histological grade are independent prognostic factors in PDACs patients [113]. Radiomics was used to discriminate between histological grades in patients with pancreatic cancer. A recent study showed that CT-based radiomics may become a new noninvasive method to predict the histological grades of PDAC preoperatively, with an excellent AUC of 0.961 and 0.910 in the training and test data sets, respectively [114]. Besides, the CT radiomics could help differentiate R0 from R1 (a resection margin without cancer cells in 1 mm is recognized as R0; a resection margin with cancer cells in 1 mm is recognized as R1) before surgery, which was of importance for making surgical decisions and predicting prognosis [115].

Studies regarding the application of radiomics in predicting pancreatic cancer survival models were also reported [116–120]. In addition, CT-based radiomic methods were used to select an appropriate candidate for irradiation stents in patients with unresectable pancreatic cancer or predict pancreatic fistula operatively in patients who would receive pancreaticoduodenectomy [121, 122] (Table 3).

We reviewed these studies of radiomics in patients with pancreatic neuroendocrine tumors in this part. The applications of radiomics included the prediction of tumor subtypes, metastasis, treatment response, and prognosis. Although these explorations are still at the preliminary level, their future developments are expected to path the way for more robust studies, which could one day eventually find their applications in clinical practice [123].

### 3.5. Adrenal Tumors

Pheochromocytoma (PHEO) is a type of rare neuroendocrine tumor that originated from chromaffin cells of the adrenal medulla. Patients with PHEO may suffer from severe cardiovascular and cerebrovascular diseases. Therefore, early diagnosis and treatment are of vital importance in PHEO patients. It is easy to diagnose PHEO if there exist definite diagnostic features. However, for asymptomatic pheochromocytoma, it is still difficult for radiologists and surgeons to distinguish some pheochromocytoma from lipid-poor adenomas (LPAs, those with CT attenuation values over 10 HU on unenhanced CT) because their imaging features are highly overlapping. CT-based radiomic methods had been shown to be effective in differentiating between asymptomatic pheochromocytoma and LPAs [124, 125].

Radiomics was also used to assess the localization of primary aldosteronism [126]. Although the applications of radiomics in the adrenal gland are rarely reported, it may be widely used not only in the diagnosis of tumors but also in the prediction of metastasis and prognosis in the future due to its noninvasiveness and repeatability.

### 3.6. Ovarian Tumors

#### 3.6.1. Tumor Subtypes Classification

Radiomics is widely applied in the classification of ovarian tumors. According to the American Cancer Society 2017, ovarian cancer was the deadliest of all gynecologic tumors. The reason for the poor prognosis is the lack of technology for early screening and diagnosis [127, 128]. Ultrasound has become the main examination for assessing ovarian pathology and has an excellent performance in preoperatively distinguishing benign and malignant ovarian tumors [129]. Martínez-Más et al. evaluated the classification of ovarian tumors by using ultrasound radiomics, achieving an excellent accuracy of more than 85% [130]. Nougaret et al.'s study showed that CT radiomic features of serous borderline tumors were distinct from low-grade serous carcinomas [131]. Optical coherence tomography (OCT) showed great potential in diagnosing diseases and classifying tissues [132]. Sawyer et al. developed a three-dimensional (3D) texture analysis of OCT images in mouse ovarian tissues. The results showed that the 3D texture analysis of OCT was mostly effective for differentiating tissue types with an accuracy of 78.6% [132]. Similarly, St-Pierre et al. performed a study basing on OCT and showed an accuracy of more than 70% in the detection of high-grade serous, endometroid, and clear cells cancers [133]. Wen et al. explored texture analysis basing on second harmonic generation (SHG) images in the application of classifying ovarian cancer, achieving high accuracy on distinguishing normal ovarian tissue from high-grade cancer tissue [134, 135].

#### 3.6.2. Metastasis and Treatment Response

It is important to differentiate localized from metastatic ovarian cancer because the tumor staging determines patient management. Pouli et al. used SHG radiomic methods to identify ovarian cancer peritoneal metastases, revealing that metastatic tissue image features were distinct to that of healthy tissues with excellent accuracy, sensitivity, and specificity of 97.5%, 100%, and 96.6%, respectively [136].

Effective chemotherapy after operation helps improve the survival rate of metastatic ovarian cancer patients, but the response to chemotherapy is variable in individual patients and how to choose candidates for chemotherapy at an early-stage remains critical. Danala et al. used CT-based radiomic methods to predict responses of ovarian cancer patients to chemotherapy. The result found that the model's AUC was higher than 0.8 when using two corresponding image markers. It also revealed that radiomic features difference computed between pre- and post-therapy CT images performed higher prediction accuracy [137]. Basing on CT radiomics, Zargari et al. evaluated a similar study and generated an AUC of 0.86 [138].

#### 3.6.3. Prognosis

After treatments, most patients with early-stage ovarian cancer have a favorable prognosis, but approximately 20% of them will finally recur and die due to this disease. It is important to evaluate the prognosis preoperatively because it is related to personalized treatment and management. Lu et al. declared that CT radiomic prognostic vector (RPV) could be exploited to personalize therapy of epithelial ovarian cancer (EOC) and had the potential to apply in other cancer types [139]. According to a study by Vargas et al., CT radiomic features may predict prognosis in patients with high-grade serous ovarian cancer (HGSOC) [140]. Another recent multicenter study based on CT radiomic analysis established a radiomics signature preoperatively and validated its effectiveness to be a novel recurrence risk prognostic factor for advanced HGSOC, and the accuracy of predicting 18-month and 3-year recurrent risk were 84.1% and 88.9%, respectively [141] (Table 4).

Radiomic methods were mainly utilized for the assessment of tumor subtypes classification, metastasis, and treatment response and prognosis in patients with ovarian cancer. Although many problems need to be solved, radiomics is a potential game-changer that shifts radiology from the traditional visual analysis to more objective and automated analysis. Radiomics raises particular hope in ovarian cancer to better capture the whole disease heterogeneity and offer a new useful tool to predict tumor aggressiveness and response to therapy [142]. Future work needs to focus on the development of complete automated postprocessing methods that enable the extraction of maximal information from the images with the added challenge to demonstrate a clinical benefit in the assessment of tumor response [143].

### 3.7. Prostate Cancer

#### 3.7.1. Diagnosis and Tumor Localization

Prostate cancer (PCa) is one of the most prevalent male malignant tumors worldwide, of which the incidence is rising annually in China [144–146]. PCa has become a major health concern in families and society. Thus, early diagnosis is of important significance to patients with PCa. In a recent MRI-based study, Gleason scores >6 were considered as clinically significant (CS) PCa, and the results showed that the phenotype of CS peripheral zone PCa lesions could be predicted by using radiomic features with a maximum AUC of 0.870 [147]. Li et al. demonstrated that the MRI radiomic prediction model (with an AUC of 0.98) had a better diagnostic ability when compared with the clinical model (with an AUC of 0.79) [148].

Bagher-Ebadian et al. proposed a study to identify dominant intraprostatic lesions (DILs) in patients with PCa and declared that MRI radiomic model was adaptive to detect DILs (with an excellent AUC of 0.94) [149]. Radiomic methods including MRI and US had also been used to predict the localization of PCa, and the results demonstrated that quantitative radiomic features could be utilized to predict localization [150, 151].

#### 3.7.2. Tumor Risk Stratification and Treatment Strategies

Risk stratification for patients with PCa is critical because it is tightly associated with patients' treatment, management, and long-term survival. Chen et al. found that the MRI radiomic model had a perfect AUC of more than 0.98 to distinguish PCa from non-PCa patients and had an excellent AUC of more than 0.86 to assess the tumor aggressiveness [144]. Several similar studies had also proved that radiomic features had the potential to predict risk stratification of PCa [152–155].

Radiation therapy (RT) is one of the major treatments for patients with localized PCa. Basing on MRI radiomics, Shiradkar et al. designed a study aiming to make personalized targeted focal treatment plans, and the results found that the focal treatment plans were decreased in dose to the organs at risk and an increased dose to the cancerous lesions [156].

#### 3.7.3. Prognosis

The Gleason score is commonly used in clinical both as a prognostic factor and to determine patient treatment in patients with PCa [157]. Toivonen et al. tried to explore whether MRI radiomic features can improve noninvasive PCa characterization and found that radiomic features had a good classification performance for Gleason score of PCa patients with a maximum AUC of 0.88 [158]. Basing on MRI radiomic features, Penzias et al. conducted a similar study and aimed to distinguish different Gleason grades of PCa, achieving an AUC of 0.69 in Gabor texture features and 0.75 in quantitative histomorphometry features [159]. These two research studies indicated that radiomic features had the potential to predict the prognosis of PCa.

#### 3.7.4. Recurrence

Biochemical recurrence (BCR) occurs in a significant number of patients who received radical prostatectomy or radiation therapy. Therefore, it is important to predict which man will develop BCR for the early identification of personalized adjuvant therapy. In a recent study, MRI radiomic features were proved to be predictive in BCR after prostatectomy, which may help guide postoperative management [160]. Shiradkar et al. designed a preliminary study to predict BCR in patients with PCa by using pretreatment MRI radiomic features, demonstrating that radiomic features can predict PCa BCR (with a maximum AUC of 0.84) and may help identify men who would benefit from adjuvant therapy [161]. Zhong et al.'s study successfully evaluated BCR of localized PCa after radiation therapy by using MRI radiomics [162]. Bourbonne et al.'s study validated the potential of MRI radiomic models to predict BCR of high-risk PCa with an accuracy of 78% [163] (Table 5).

The applications of radiomics in PCa mainly included the prediction of diagnosis and tumor localization, tumor risk stratification and treatment strategies, recurrence, and prognosis. Radiomics is a promising new field, which allows for high-throughput analysis of imaging features extracted from existing data for PCa detection and evaluation. Therefore, the potential of radiomics for future study is immense [164].

## 4. Discussion

In this review, we briefly introduced the basic principle and workflow of radiomics and then summarized the clinical applications of radiomics in endocrine tumors, which mainly included the prediction of diagnosis, tumor subtype classification, metastasis prediction, treatment response, prognosis and recurrence, and other aspects.

Due to tumor heterogeneity, different parts of tumor have different molecular characteristics in cancer patients, and these differences are changing all the time. In order to better characterize the tumor, performing multiple tumor biopsies on the patients will cause more damage as well as more cost and psychological burden to the patients. Radiomics is expected to become a “virtual biopsy” instead of biopsy as a new golden indicator in the future because of its noninvasive properties [165].

Radiomics has brought a lot of unprecedented help to the personalized and precise medicine and patient management of the endocrine tumor in the clinic. However, there are still deficiencies, which limit the development of radiomics.

Generally, conducting radiomic research studies requires a large number of standard medical images. But the collection of imaging data is a time-consuming task, which may bring a great burden to clinicians or radiologists. In order to better apply radiomics to clinical practice in the future, these image data should be more digitized and standardized. This requires the continuous efforts of researchers around the world for a long time. Radiomics relies on the use of specialized software, which may lead to additional costs and personnel training. Few patients may result in false positives [6]. The clinical data are private in different hospitals and research institutes, which may limit the generalizability of radiomics. Thus, big data and data sharing will provide a larger platform and space for the development of radiomics, which makes radiomics better clinically applicable [166].

In current radiomic research studies, not all radiomic features can be applied to clinical prediction. For example, textures sensitive to acquisition patterns and reconstruction parameters are not recommended for malignant and benign tissue differentiation [167]. In addition, different methods of radiomic features calculation may lead to different results; tumor heterogeneity with small tumor volume cannot be accurately quantified; many radiomic features are unstable within weeks or even minutes, all of which are the current problems of radiomics [6]. Considering that the types of image acquisition, postprocessing and segmentation can affect the quality of extracted features; the correlation between features and clinical data as well as the model derived from them could also be affected. Therefore, the reproducibility and quality control of radiomic features will be an important direction in the future. Clinicians and radiologists should strive for standardization as appropriate statistical methods will minimize spurious relationships and lead to more accurate and repeatable results [168].

In the future, studies should focus on the combination of radiomics with other nonimaging biomarkers as combining different biomarkers is the most promising approach that may change clinical management. Radiogenomics, which combines radiomics with genomics, may have the potential to waive the need for invasive diagnostic procedures such as biopsy. This could be a breakthrough for future research.

Living in the present and looking forward to the future, radiomics is an emerging and rapidly developing discipline and plays an increasingly important role in precision medicine and oncology.

## Figures and Tables

**Table 1:** Different clinical applications of radiomic models (features) in PAs.

References	Case numbers	Radiomic method	Results
[20]	133	MRI	Radiomic features had promising and practical values in distinguishing pituitary adenoma from Rathke cleft cyst
[21]	235	MRI	MRI-based radiomic model could be used to predict immunohistochemical results of pituitary adenoma preoperatively
[22]	112	MRI	MRI-based radiomic features had a great potential to differentiate between nonfunctional subtypes and other subtypes pituitary adenomas preoperatively
[24]	89	MRI	Radiomics could indirectly predict tumor aggressiveness by predicting high proliferative index Ki-67 in pituitary macroadenomas
[25]	194	MRI	MRI-based radiomic method was proved to be an effective method for predicting the cavernous sinus invasion preoperatively
[28]	163	MRI	Radiomics models may help neurosurgeons predict the treatment response preoperatively and make personalized treatment strategies
[26]	400	MRI	The result indicated that early postoperative outcomes of PAs could be assessed by a radiomic approach

**Table 2:** Different clinical applications of radiomic models (features) in thyroid cancer.

References	Case numbers	Radiomic method	Results
[29]	55	^18^F-FDG-PET/CT	Radiomic features had the potential to diagnose malignant thyroid cancer
[39]	44	MRI	The textural analysis classifies thyroid nodules with high sensitivity and specificity on multi-institutional DW-MRI data sets
[34]	450	US	US-based radiomics had the potential to predict the lymph node metastasis of PTC preoperatively
[35]	189	US	The accuracy of the US-based radiomic method was much higher than that of US examination in the prediction of metastasis of PTC
[36]	43	MRI	Radiomic models may have the potential to differentiate benign from malignant nodules
[37]	527	US	Radiomic features had limited values as a noninvasive biomarker for predicting clinical aggressive behaviors
[38]	400	US	US radiomic features of the primary tumor were associated with lateral cervical lymph node status
[40]	1576	US	A CADx system using CNN-combinations may help radiologists make decisions by overcoming interobserver variability when assessing thyroid nodules on US
[41]	624	CT	Radiomic model had the potential to predict ETE preoperatively in patients with PTC
[42]	768	US	Radiomic features were significantly associated with disease-free survival

**Table 3:** Different clinical applications of radiomic models (features) in pancreatic neuroendocrine tumors.

References	Case numbers	Radiomic method	Results
[96]	164	CT	CT-based radiomic classifiers had the potential to differentiate serous cystadenoma from IPMN and MCN
[97]	38	CT	Radiomic method may more accurately predict IPMNs pathology than radiologic features considered in consensus guidelines
[98]	53	CT	Radiomics could predict the malignant potential of intraductal papillary mucinous neoplasms and had important application values in clinical decision making
[99]	260	CT	The proposed radiomic-based computer-aided diagnosis scheme could increase preoperative diagnostic accuracy and assist clinicians in making accurate management decisions
[100]	78	CT	Radiomics made a contribution to the differentiation of pancreatic serous cystadenomas and mucinous cystadenomas
[101]	225	CT	Radiomic features were independently and positively associated with the risk of LN metastasis in PDAC
[102]	159	CT	CT radiomic signature could be conveniently used for preoperative prediction of lymph node metastasis in patients with PDAC
[105]	20	CT	CT radiomic features may be potentially used for early assessment of treatment response and stratification for therapeutic intensification
[106]	90	CT	Radiomics may develop into a biomarker for early prediction of treatment response
[107]	74	CT	Overall survival and recurrence could be better predicted with models based on radiomic features than with those based on clinical features for pancreatic cancer
[108]	24	CT	Combining radiomics with CA19-9 could improve the ability to predict posttreatment responses
[112]	Not mentioned	MRI	Radiomics could be used as an imaging biomarker for early immunotherapy response assessment in a KPC transgenic mouse model of PDAC
[114]	301	CT	CT radiomic signature showed moderate predictive accuracy for differentiating low-grade from high-grade PDAC and should become a new noninvasive method for the preoperative prediction of histological grades of PDAC
[115]	86	CT	Radiomics was rewarding for the aided diagnosis of R0 and R1. Texture features could potentially enhance physicians' diagnostic ability
[116]	88	CT	CT radiomics could be used for predicting the prognosis in pancreas head cancer patients who underwent curative resection
[117]	63	MRI	MRI-based radiomic features were associated with overall survival in patients with pancreatic cancer
[118]	132	MRI	Radiomic models had the potential to predict tumor subtypes and overall survival in PDAC
[119]	100	CT	A CT-based radiomic signature was correlated with overall survival and local control after stereotactic body radiation therapy and allowed to identify low and high-risk groups of patients
[120]	98	CT	The proposed survival model outperforms Cox proportional hazard model-based radiomic pipeline in PDAC prognosis
[121]	106	CT	Radiomics was assisted in selecting an appropriate candidate for irradiation stents in patients with unresectable pancreatic cancer
[122]	117	CT	Radiomics had the potential to predict pancreatic fistula operatively in patients who would receive pancreaticoduodenectomy

**Table 4:** Different clinical applications of radiomics in ovarian tumors.

References	Case numbers	Radiomic method	Results
[130]	187	US	US-based radiomics could be efficiently used for developing the classification stage in ovarian tumor
[131]	59	CT	CT features of serous borderline tumors were distinct from low-grade serous carcinomas
[132]	Not mentioned	OCT	3D texture analysis of OCT was useful for quantitatively characterizing ovarian tissue
[133]	38	OCT	OCT-based radiomics had the potential to classify different subtypes of ovarian tissue
[134]	10	SHG	SHG texture analysis had the potential for ovarian cancer classification
[135]	10	SHG	3D SHG texture analysis achieved high accuracy for classifying high-grade cancer tissue and normal ovarian tissue
[136]	8	SHG	Metastatic tissue images features were distinct from that of healthy tissues
[137]	91	CT	CT-based radiomics had the potential to predict responses of ovarian cancer patients to chemotherapy
[138]	120	CT	CT-based radiomic features computed from both spatial and frequency domains had a reliable prediction ability of tumor response to postsurgical chemotherapy
[139]	364	CT	Radiomic prognostic vector (RPV) could be exploited to personalized therapy of epithelial ovarian cancer (EOC) and had the potential to apply in other cancer types
[140]	38	CT	Quantitative metrics noninvasively capturing spatial intersite heterogeneity may predict outcomes in patients with HGSOC
[141]	142	CT	Radiomic signature was potential prognostic markers that may allow for individualized evaluation of patients with advanced HGSOC

**Table 5:** Different clinical applications of radiomic models (features) in prostate cancer.

References	Case numbers	Radiomic method	Results
[147]	206	MRI	The phenotype of clinically significant peripheral zone PCa lesions could be predicted by using radiomic features
[148]	381	MRI	Radiomic prediction model had an improved diagnostic ability when compared with the clinical model
[149]	117	MRI	Radiomic model was adaptive to detect dominant intraprostatic lesions in patients with PCa
[150]	30	MRI	Quantitative radiomic features based on MRI radiomics could be utilized to predict the localization of PCa
[151]	50	US	Quantitative radiomic features based on US radiomics could be utilized to predict the localization of PCa
[144]	381	MRI	MRI-based radiomic models had a reliable ability to distinguish PCa with non-PCa patients as well as assess the tumor aggressiveness
[153]	73	MRI	Radiomic features had the potential to predict risk stratification of PCa
[156]	23	MRI	The focal treatment plans formed by using the framework were decreased in dosage to the organs at risk and a boosted dose delivered to the cancerous lesions
[158]	62	MRI	Radiomic features had good classification performance for Gleason score of patients in PCa
[159]	71	MRI	Radiomic features had the potential to predict the prognosis of PCa
[160]	107	MRI	Radiomic features were predictive of biochemical recurrence after prostatectomy in PCa
[161]	120	MRI	Radiomic features can be predictive of PCa BCR and may help identify men who would benefit from adjuvant therapy
[162]	91	MRI	MRI-based radiomics could predict BCR of localized PCa after radiation therapy
[163]	195	MRI	MRI-based radiomic models had the potential to predict BCR of high-risk PCa

## Data Availability

No data were used to support this study.
